# Comparison of Nailfold Videocapillaroscopy with Retinal and Choroidal Vascular Parameters in Patients with Central Serous Chorioretinopathy

**DOI:** 10.3390/jcm12144817

**Published:** 2023-07-21

**Authors:** Małgorzata Latalska, Joanna Bartosińska, Sławomir Dresler, Mario Damiano Toro, Dorota Krasowska, Robert Rejdak

**Affiliations:** 1 Department of General and Pediatric Ophthalmology, Medical University of Lublin, 20-079 Lublin, Poland; mariodamiano.toro@unina.it (M.D.T.); robert.rejdak@um.lub.pl (R.R.); 2 Department of Cosmetology and Aesthetic Medicine, Medical University of Lublin, 20-093 Lublin, Poland; jbartosinski@gmail.com; 3 Department of Analytical Chemistry, Medical University of Lublin, Chodźki 4a, 20-093 Lublin, Poland; 4 Department of Plant Physiology and Biophysics, Institute of Biological Sciences, Maria Curie-Skłodowska University, Akademicka 19, 20-033 Lublin, Poland; 5 Eye Clinic, Public Health Department, University of Naples Federico II, Via S. Pansini 5, 80133 Naples, Italy; 6 Department of Dermatology, Venereology and Pediatric Dermatology, Medical University of Lublin, 20-059 Lublin, Poland; dorotakrasowska@umlub.pl

**Keywords:** nailfold videocapillaroscopy, central serous chorioretinopathy, microcirculation, choroid, vessel density

## Abstract

Aim: This study seeks to evaluate the results of nailfold videocapillaroscopies (NVCs) among patients with central serous chorioretinopathy (CSC) and their correlation with the choroid and retinal parameters. Material and Methods: The examined group included 152 patients with acute, recurrent, chronic and neovascular CSC (34 F, 118 M, mean age 45.9 ± 8.9) and 41 healthy controls (12 F, 29 M, mean age 47 ± 11.5). The NVC examination, ophthalmoscopy, angio-OCT and OCT were performed. In addition, the medical history regarding chronic general disorders and known risk factors were recorded. Results: Abnormal NVC patterns and the dilated apical part of capillaries were found only in CSC patients (*p* = 0.000). Neoangiogenesis was observed in 25 acute (58.14%), 22 recurrent (42.31%), 16 chronic (36.36%) and 5 neovascular patients (45.45%) and 2 control subjects (4.88%) (*p* = 0.000). Glomerular capillaries were found in 8 acute (18.6%), 17 recurrent (31.48%), 25 chronic (56.82%) and 8 neovascular patients (72.73%) (*p* = 0.000). Meandering capillaries were more common in acute and recurrent CSC and glomerular capillaries were more common in chronic and aneurysmal dilations in neovascular CSC. Conclusions: The observed digital microcirculation abnormalities in patients with CSC, such as dilation, meandering, tortuosity and glomerular, may confirm systemic micro-vasculopathy. The potential role of the NVC examination in assessing the CSC prognosis requires further evaluation.

## 1. Introduction

Central serous chorioretinopathy (CSC) is a common disorder that typically affects working-age males, with a male-to-female ratio of approximately 3:1 [1]. CSC is characterized by the accumulation of serous subretinal fluid (SRF) caused by dysfunction of the retinal pigment epithelium (RPE) due to the hyperpermeability and thickening of the underlying choroid [1]. 

Although von Graefe described the condition in 1866, the cause remains unknown [2]. Recently, many studies explored microcirculatory disturbances in the choroid of eyes with CSC [3,4,5,6]. Bacci et al. proposed that asymmetric vortex veins revealed in CSC may cause venous overload, especially in the macula region [7]. Venous congestion may lead to the formation of pachyvessels and pachychoroids [8]. In addition, congestion of the vortex veins, especially the one draining the macula, might contribute to the engorgement of vessels at the Haller layer. Panga et al. observed the dilation of one or more vortex veins in 83.3% of CSC eyes [6]. In addition, Brinks et al. found abnormal choroidal venous anastomoses within the vortex vein system, which might lead to congestion of blood flow in the choriocapillaris and the breakdown of the RPE barrier [4].

Moreover, recent studies have shown increased anterior and posterior scleral thickness in CSC patients compared to controls. These results may indicate that scleral thickening may mechanically block the choroidal venous outflow [6,7,8]. These changes may result in choriocapillaris ischemia because the choriocapillaris obtains direct pressure from both sides by branches of the short posterior ciliary arteries and by collecting venules of the congested vortex vein [8]. Compression of the choriocapillaris may cause a reduction in the vascular flow area, while Sattler’s and Haller’s layers reveal increased blood flow [9]. Early ischemia was proved by reduced choroidal capillary density at the leakage point in acute CSC and tended to regenerate with recovery [10]. Moreover, Spaide et al. proposed a relation between CSC and choriocapillaris abnormalities independent of the pachychoroid [11].

Previous studies evaluating nailfold videocapillaroscopy revealed additional digital microcirculatory disturbances at the nail cuticle level of CSC patients, suggesting that CSC might be a part of systemic micro-vasculopathy [12,13].

Nailfold videocapillaroscopy (NVC) is one of the best diagnostic imaging techniques for studying microcirculation in vivo. It is a non-invasive, repeatable, simple and inexpensive method that directly permits microcirculation assessment [14]. It is widely used in diagnosing microvascular pathologies in dermatologic and rheumatologic conditions, as well as in diabetes mellitus (DM) and arterial hypertension. Furthermore, NVC may aid in the early diagnosis of connective tissue disorders and the differential diagnosis of primary Raynaud phenomenon (RP) from secondary RP due to systemic scleroderma (SSc) and mixed connective tissue disease (MCTD) [15]. Compared to standard nailfold capillaroscopy, it has advantages like real-time control of the image obtained, image storage and reproduction precision, and advanced image analysis and measuring features. A further advantage is the presence of a contact probe with polarized light microscopy, allowing a more straightforward observation of the skin surface [15]. Therefore, NVC is becoming increasingly popular in ophthalmology, especially in assessing microcirculation in glaucoma [16]. 

This study aimed to evaluate the results of nailfold videocapillaroscopic examination in patients with different types of CSC in comparison to age-matched healthy volunteers. The secondary study’s goal was to also assess the relationship between the results of this examination with vascular retinal and choroid parameters, clinical status and observed risk factors among the CSC groups.

## 2. Materials and Methods

### 2.1. The Study Groups

The study group consisted of 152 patients suffering from CSC treated in a single center in 2018–2022 and 41 healthy gender and age-matched controls (HCs). Researchers obtained written informed consent from all the participants before their enrollment in the study. The study was performed in accordance with the tenets of the Declaration of Helsinki and the design was approved by the local ethical committee at the Medical University of Lublin (KE-0254/291/2018). The current study was a continuation and extension of the previous study [13].

**Figure 1:**
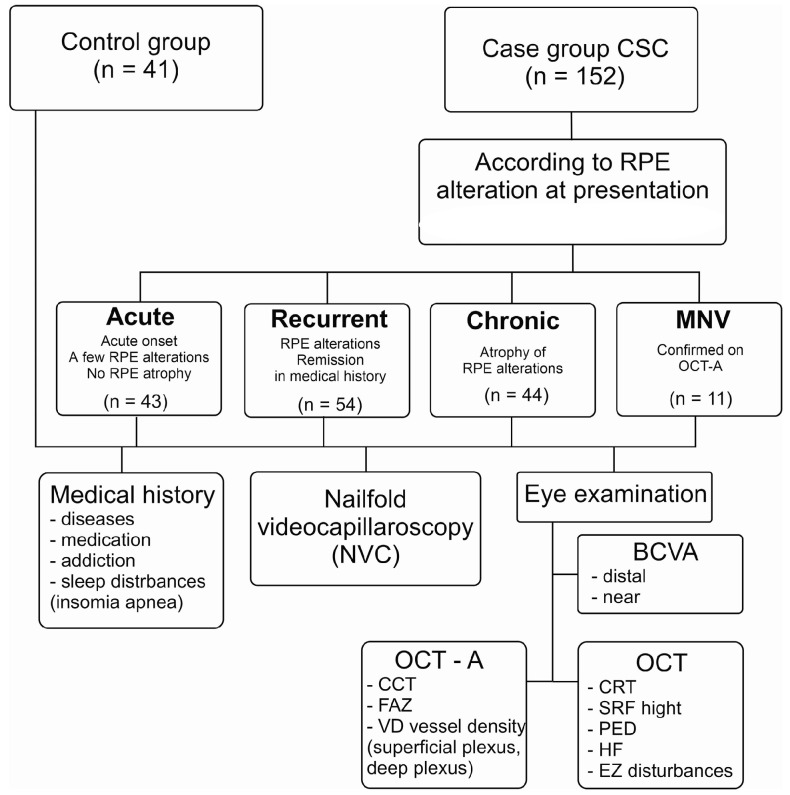
Study design Flow Chart. CSC—central serous chorioretinopathy, RPE—retinal pigment epithelium, OCT—optical coherence tomography, BCVA—best corrected visual acuity, CCT—central choroidal thickness, FAZ—foveal avascular zone, CRT—central retinal thickness, SRF—subretinal fluid, PED—pigment epithelium detachment, HF—hyper-reflective foci, EZ—ellipsoid zone.

In the study, we enrolled 34 female and 118 male CSC patients and divided them into acute (aCSC), recurrent (rCSC), chronic (cCSC) and macular neovascularization groups (nCSC) based on clinical and multimodal imaging findings (mean age of aCSC 46.30 ± 0.9, rCSC 46.61 ± 0.9, cCSC 47.5 ± 1.01 and nCSC 47.45 ± 2.08). The study design Flow Chart is presented in Figure 1. Concerning age and sex, there were no significant differences between all CSC groups and controls (*p* = 0.901, *p* = 0.988). 

The inclusion criterion for the CSC group was an active CSC in one eye, defined as a serous detachment of the neuroretina (confirmed after clinical and spectral domain optical coherence tomography examination—OCT). The fellow eye was also assessed.

Patients with an active CSC were divided into subgroups according to the following criteria:

1/acute CSC (aCSC) defined as an acute-onset, serous neuroretinal detachment in the macula with a few possible RPE alterations inside the area of detached retina;

2/recurrent CSC (rCSC) defined as a subsequent episode of acute CSC based on the medical history and presentation of RPE alterations in the location unrelated to the current serous detachment, indicating previous episodes;

3/chronic CSC (cCSC) defined as a history of persistent visual disturbances and serous neuroretinal detachment with/without PED, associated with atrophy of the RPE and outer retina layers, including gravitational tract;

4/neovascular CSC (nCSC) defined as a serous neuroretinal detachment complicated by macular neovascularization (MNV) confirmed by OCT—A examination.

The exclusion criteria for the CSC group were as follows: 1/autoimmune diseases such as systemic sclerosis (SSc) and systemic lupus erythematosus (SLE) due to their significant impact on NVC scores; 2/general vascular and neoplastic disorders (e.g., peripheral artery disease, abdominal aortic aneurysm, carotid artery disease, pulmonary embolism, chronic venous insufficiency and anemia); 3/history of anti-cancer treatment; 4/debilitating conditions such as chronic alcoholism or drug addiction; 5/concurrent ocular and retinal disease affecting visual acuity, including diabetic retinopathy, age-related macular degeneration, vitreomacular disorders, presence of profound RPE atrophy in the fovea and cystic degeneration in the macula on structural OCT; 6/myopia exceeding six diopters due to its significant impact on choroid thickness.

As described later, the HC group was examined to exclude any ocular diseases. That group included 12 female and 29 male (mean age 47.12 ± 0.76) healthy volunteers. 

### 2.2. Ophthalmologic Examination 

All participants (CSC patients and HCs) underwent best corrected distal (BCDVA) and near (BCNVA) visual acuity examinations and fundus ophthalmoscopy. Ultra-widefield color and autofluorescence fundus photography were performed with Optos California (Optos, Inc., Marlborough, MA, USA). Visual acuity was tested using Snellen charts at a distance of 5 m in a room with standardized illumination. BCDVA was measured with the best correction obtained with subjective refraction.

**Figure 2:**
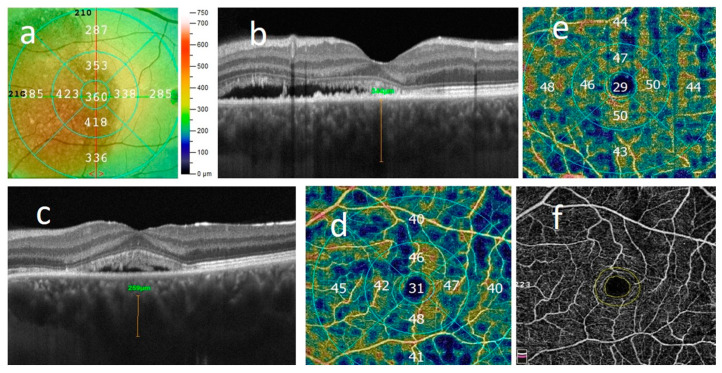
Multimodal imaging features: (**a**) CRT—central retinal thickness; (**b**) CCT—central choroidal thickness; (**c**) diameter of pachyvessel; (**d**) vessel density of superficial plexus; (**e**) vessel density of deep plexus; (**f**) FAZ—foveal avascular zone.

Spectral domain OCT and OCT-angiography (OCT-A) were performed using Angio Retina QuickVue, Angio Retina and Cross Line scan (Algoritm Version A2017,1,0,151, Optovue, Inc., Fremont, CA USA) after pupil dilation. Scans with the highest resolution were obtained in the central 3 × 3 and 6 × 6 mm area, centered on the foveola. The OCT images of all subjects were reviewed for qualitative features and analyzed for quantitative measures of central retinal thickness (CRT), subfoveal subretinal fluid height (SRF), pigment epithelium detachment (PED), ellipsoid zone disruption (EZ), RPE alteration (disruption or atrophy of RPE layer) and presence of intraretinal hyper-reflective foci (HF). Central retinal thickness (CRT) was automatically measured in the central 1.0 mm circle of the EDTRS grid from ILM to the outer boundary of the RPE, uniformly to previous studies in order to compare results (Figure 2a). SRF height was measured in the fovea as a distance between the top of the SRF and the RPE–Bruch’s membrane complex.

Central choroid thickness (CCT) was measured from the outer hyper-reflective line corresponding to RPE–Bruch’s membrane layer to the inner hyper-reflective surface of the sclera (Figure 2b). The diameter of the largest visible hyper-reflective lumen of the choroid venous (pachyvessel) found on a horizontal Cross-link scan was measured by the method described by Yang et al. (Figure 2c). According to Spaide et al., those pachyvessels are formed by the intervortex venous anastomoses primarily located in the central macula in eyes with CSC [5]. Therefore, we were looking for the broadest lumen of the choroid vessel on a scan crossing the fovea.

The superficial capillary plexus was detected automatically between the internal limiting membrane (ILM) and the inner plexiform layer (IPL), whereas the deep capillary plexus was between the IPL and the outer plexiform layer (OPL). Superficial and deep vessel density (VD) were measured on 6 × 6 mm scans in the whole image, in the central 1 mm area of the fovea, surrounding the 3 mm diameter inner ring (parafovea), and in the full 3 × 3 mm circular region (full) based on the ETDRS grid using the AngioAnalytics software OptoVue system (Version 2017.1.0,151) (Figure 2d,e). The vessel density (VD) was defined as the proportion of the vessel area with the blood flow over the total area measured.

The foveal avascular zone (FAZ) was evaluated using the built-in AngioAnalytics software (Version 2017.1.0,151) OptoVue system with an automated segmentation algorithm (Figure 2f). FAZ was defined as a region within the fovea centralis at the center of the retina that is devoid of retinal blood vessels.

Researchers assessed the known risk factors for CSC like smoking, stress, steroid use, xylometazoline and phosphodiesterase inhibitor intake, diabetes mellitus, autoimmunological diseases, insomnia and obstructive sleep apnea using reports.

### 2.3. Nailfold Videocapillaroscopy in the CSC Patients and HCs

In all CSC patients and HCs, an experienced dermatologist blinded to clinical data (J.B.) performed the NVC with a videocapillaroscope VideoCap 3.0 (DSMedica) at 200× magnification. 

Before the NVC examination, every patient reported, if any, the symptoms of hands freezing.

The examination was carried out according to a standard protocol. All participants had to fast, avoid smoking, avoid drinking alcohol and caffeinated drinks and avoid taking any drugs that could affect the circulatory system 24 h before the examination. They were also instructed not to remove fingernail cuticles for one month before the test. The examination was performed at the room temperature of 21–23 °C, and then patients waited for 15–20 min. The examinator placed a drop of immersion oil on each examined finger cuticle to facilitate visibility. During NVC the patient was sitting with his/her hands at the heart level.

The nailfold capillaries of the second to fifth fingers on both hands were examined. In the NVC the following parameters were evaluated: capillary distribution (i.e., capillary architecture), capillary length, capillary morphology (i.e., meandering capillaries, coiled capillaries, tortuous capillaries, bushy-ramified capillaries and angiogenesis), capillary diameter (i.e., dilated capillaries (>20 µm), dilated apical part of capillaries, capillary enlargement (30–50 µm), megacapillaries (>50 µm), capillary density (normal 9–14 capillaries per mm), the presence of microhemorrhages and microaneurysms, and visibility of subpapillary venular plexus.

Normal capillary architecture refers to a homogeneous distribution of capillaries arranged in parallel in the distal row of the nailfold. Disorganization of the capillary architecture was defined as irregular capillary distribution, orientation and morphology [14].

After the detailed description of the nailfold capillaries, the results were interpreted as normal or abnormal NVC patterns. Normal NVC pattern was described as follows: normal capillary architecture, normal capillary density (9–14 capillaries per millimeter) and the presence of ≤2 tortuous/coiled capillaries per millimeter and/or ≤2 meandering capillaries per millimeter and/or ≤2 bizarre loops per millimeter and/or ≤1 ramified capillary per millimeter. 

An abnormal NVC pattern was defined as follows: disorganization of the capillary architecture and/or >2 tortuous/coiled capillaries per millimeter and/or >2 meandering capillaries per millimeter and/or >2 bizarre loops per millimeter and/or >1 ramified capillary per millimeter and/or >2 dilated capillaries (diameter 20–50 µm) and/or decreased capillary density and/or the presence of at least one megacapillary, broken capillary, ramified capillary and fresh microhemorrhage.

All results were compared to the “scleroderma pattern” to exclude patients suspected of undiagnosed SSc [9,10]. 

### 2.4. Statistical Analysis

Categorical variables were subjected by the Pearson’s chi-squared test (*p* < 0.05) and are presented as a number of individuals and percentage frequencies. Additionally, multiple correspondence analysis was performed to show relationships between categorical variables. The Shapiro–Wilk test was used to evaluate the normality of continuous data (*p* < 0.05). Normally distributed variables were analyzed using a one-way analysis of variance (ANOVA) followed by Tukey’s post hoc test (*p* < 0.05), while for non-normally distributed variables a Kruskal–Wallis test was applied (*p* < 0.05). The relationship between variables was estimated using Spearman’s correlation coefficients. The logistic regression creator module of the Statistica software (Statistica ver. 13.3.03. (Tibco Software Inc. Palo Alto, CA, USA)) was used to provide the regression coefficient and odds ratio. Only variables with a *p* value < 0.09 in the univariate model and variables with a linear relationship between the log odds and the predictor variables were considered. Additionally, to avoid multicollinearity, only not correlated variables were included in the model. To evaluate the goodness of fit of the logistic regression model fit statistics were calculated, including Akaike information criterion (AIC), Hosmer–Lemeshow goodness-of-fit test, the Cox–Snell R2 and Nagelkerka R2. The statistical analysis was performed using Statistica ver. 13.3 software (TIBCO Sofware Inc., Palo Alto, CA, USA). 

## 3. Results

### 3.1. Primary Outcomes

#### 3.1.1. Demographic and Clinical Data

The demographic and clinical data of CSC and HC groups are presented in Table 1.

#### 3.1.2. Comparison of NVC Parameters between Acute, Recurrent, Chronic and Neovascular CSC Patients and HCs

We found abnormal NVC patterns only in CSC patients: 24 aCSC (55.81%), 22 rCSC (40.74%), 15 cCSC (34.09%) and 7 nCSC (63.64%) (*p* = 0.000). Ramified capillaries and neoangiogenesis were observed in 25 aCSC patients (58.14%), 22 rCSC (42.31%), 16 cCSC (36.36%), 5 nCSC (45.45%) and only 2 HCs (4.88%) (*p* = 0.000). Glomerular capillaries were found in 8 aCSC patients (18.6%), 17 rCSC (31.48%), 25 cCSC (56.82%), 8 nCSC (72.73%) and only 1 HC (2.44%) (*p* = 0.000). The dilated apical part of capillaries was found only in CSC patients (*p* = 0.000): 18 aCSC (41.86%), 17 rCSC (31.48%), 12 cCSC (27.27%) and 1 nCSC (9.09%). Meandering capillaries were observed in 18 aCSC patients (41.86%), 22 rCSC (40.74%), 8 cCSC (18.18%) and 4 nCSC (36.36%) in comparison to 3 HC (7.32%) (*p* = 0.035). Hands freezing was reported only by CSC patients, except for cCSC (*p* = 0.000): 15 aCSC (34.88%), 8 rCSC (14.81%) and 4 nCSC (36.36%).

Furthermore, glomerular capillaries were significantly more common in cCSC patients than aCSC and rCSC (*p* = 0.000 and *p* = 0.011). There was no significant difference between cCSC and nCSC (*p* = 0.335). In nCSC patients, aneurysmal dilatations were significantly more common than in aCSC and cCSC patients (*p* = 0.009 and *p* = 0.008). There was no difference between nCSC and rCSC (*p* = 0.093). Moreover, in aCSC cases, compared to cCSC, an abnormal NVC pattern, hands freezing, ramified capillaries and neoangiogenesis, and meandering capillaries were significantly more common (*p* = 0.041, *p* = 0.000, *p* = 0.04 and *p* = 0.015, respectively). In aCSC patients, hands freezing was significantly more common than in rCSC patients (*p* = 0.023). In comparison to nCSC those patients had more frequently dilated apical parts of capillaries and less common aneurysmal dilatations (*p* = 0.042 and *p* = 0.009). In cases of aCSC and rCSC, meandering capillaries were significantly more common than in cases of cCSC (*p* = 0.015, *p* = 0.015). 

The diameter of nailfold capillaries was significantly higher for all CSC groups compared to HCs (*p* = 0.000). The mean diameter for HCs was 12.09 ± 4.17 µm, while in aCSC was 23.81 ± 8.15 µm, in cCSC was 17.41 ± 8.68 µm, in rCSC was 15.97 ± 7.25 µm and in nCSC was 18.81 ± 7.40 µm. Among the CSC groups, the diameter was significantly smaller for cCSC and rCSC than aCSC (*p* = 0.003 and *p* = 0.002). There was no significant difference between aCSC and nCSC (*p* = 1.0). 

**Figure 3:**
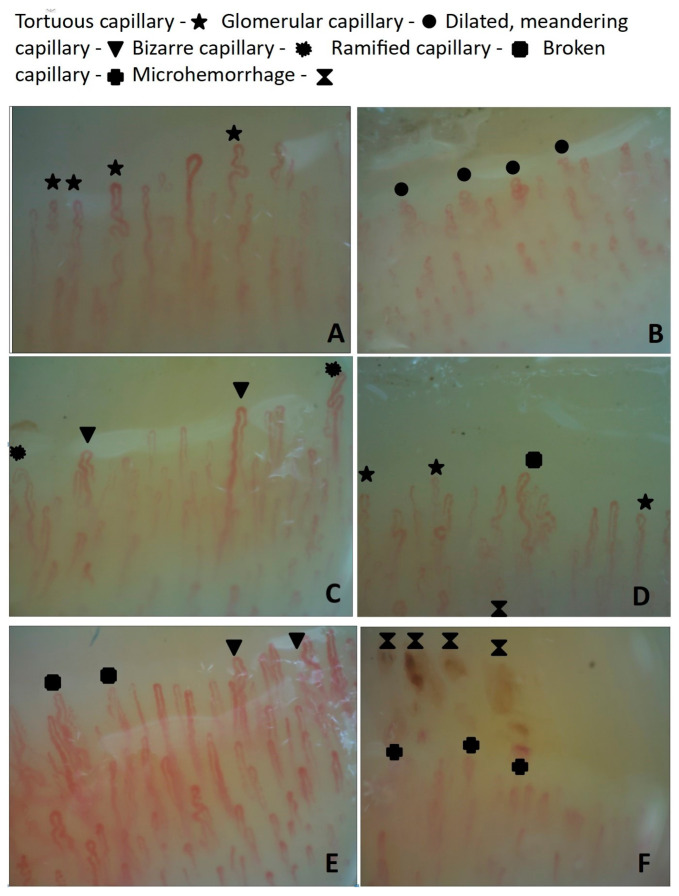
Capillary changes in patients with CSC: (**A**) tortuous capillaries; (**B**) glomerular capillaries; (**C**) dilated, meandering and bizarre capillaries; (**D**) tortuous, ramified capillaries and neoangiogenesis; (**E**) dilated, meandering and ramified capillaries; (**F**) abnormal distribution, broken capillaries and microhemorrhages.

Capillary changes in patients with central serous chorioretinopathy are presented in Figure 3. 

**Figure 4:**
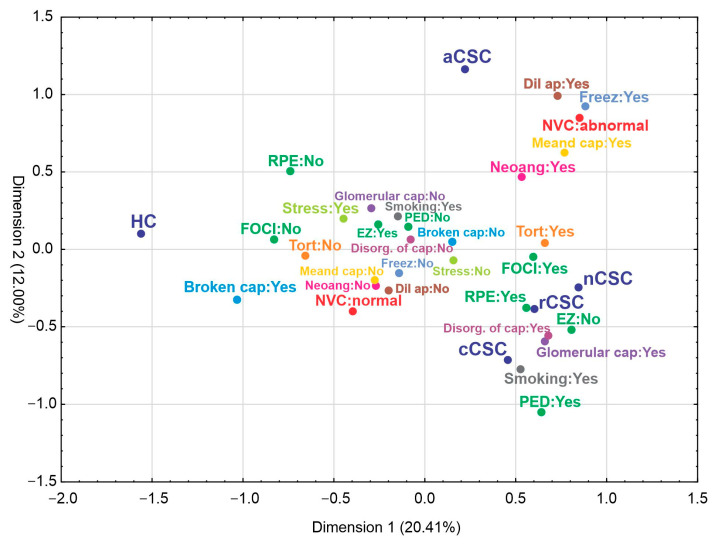
Two-dimensional correspondence plot using multiple correspondence analysis. FOCI—HF (hyper-reflective foci), RPE—RPE alteration, PED—pigment epithelium detachment, Meand. cap—meandering capillaries, Neoang—ramified capillaries and neoangiogenesis, Tort—tortuous capillaries, Glomerular cap—glomerular capillaries, Disor. of cap—disorganization of capillaries, Dil. ap—dilated apical part of capillaries, NVC abnormal—abnormal NVC pattern, EZ:No—EZ disturbances, Freez—hands freezing.

A multiple correspondence analysis was used to explore multivariate relationships within the data. The separated parameters based on types of disease are presented in Figure 4. The first two identified dimensions explained over 32% of total inertia. Based on the variable factor score map the first dimension facilitated separation control from CSC. In turn, the second dimension separated aCSC from the rest of the CSCs. It was showed that CSC patients, contrary to HCs, were associated with the presence of HF, RPE alteration, PED, meandering capillaries, ramified capillaries and neoangiogenesis, tortuous capillaries, glomerular capillaries, the disorganization of capillaries, the dilated apical part of capillaries or abnormal NVC pattern, EZ disturbances, hands freezing and smoking. 

### 3.2. Secondary Outcomes

#### 3.2.1. Correlations between NVC Results and Retinal Vessel Density (VD) of Superficial and Deep Plexus, FAZ, Central Choroid Thickness and Diameter of Choroid Anastomotic Vessel Lumen in CSC Patients

**Figure 5:**
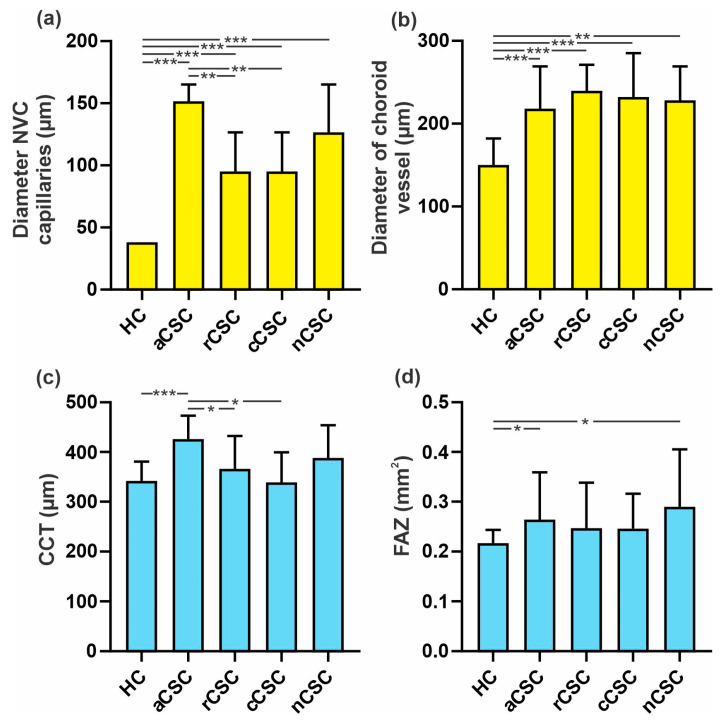
Comparison of diameter of NVC capillaries (**a**) and choroid pachyvessel (**b**), CCT (**c**) and FAZ (**d**) in CSC groups and control group (HCs): * *p* < 0.05, ** *p* < 0.01, *** *p* < 0.001.

There was a significant correlation between the diameter of nailfold capillaries and CCT of the affected and fellow eye and the FAZ and diameter of the pachyvessel of the affected eye (*p* = 0.000, *p* = 0.002, *p* = 0.001 and *p* = 0.009, respectively). The abnormal NVC pattern was significantly correlated with the diameter of the pachyvessel lumen (*p* = 0.006). Ramified capillaries and neoangiogenesis correlated considerably with CCT, the FAZ, the VD of the deep plexus of the fovea of the affected eye and the deep plexus of the inferior and nasal sectors of the fellow eye, and the diameter of the pachyvessel lumen (*p* = 0.000, *p* = 0.032, *p* = 0.001, *p* = 0.04, *p* = 0.001 and *p* = 0.000, respectively). Tortuous capillaries were significantly correlated with the CCT of both eyes, the VD of the deep plexus of the fovea, and the diameter of the pachyvessel lumen of the affected eye and the inferior and nasal sectors of the fellow eye (*p* = 0.004, *p* = 0.039, *p* = 0.045, *p* = 0.000 and *p* = 0.028, respectively). Glomerular capillaries were significantly correlated with the FAZ, the VD of the superficial and deep plexus of the fovea and parafovea sectors of the affected eye, and the diameter of the pachyvessel lumen (*p* = 0.018, *p* = 0.000, *p* = 0.000, *p* = 0.002, *p* = 0.025 and *p* = 0.002, respectively). We observed a significant correlation between the dilated apical part of capillaries and the VD of the superficial and deep plexus of the fovea and the deep plexus of the inferior and temporal sectors in the affected eye (*p* = 0.000, *p* = 0.001, *p* = 0.005 and *p* = 0.008, respectively). We also found a significant correlation between meandering capillaries and the diameter of the pachyvessel lumen (*p* = 0.000). Freezing hands reported by patients was significantly correlated with the FAZ and the VD of the superficial and deep complex of the parafovea and the deep plexus of the superior, inferior and temporal sectors of the affected eye (*p* = 0.002, *p* = 0.006, *p* = 0.006, *p* = 0.037, *p* = 0.014 and *p* = 0.014, respectively). Hands freezing was also significantly correlated with parameters of the fellow eye: the FAZ and the VD of the superficial plexus of the fovea, parafovea, superior, inferior, temporal and nasal sectors (*p* = 0.000, *p* = 0.021, *p* = 0.007, *p* = 0.001, *p* = 0.002, *p* = 0.040 and *p* = 0.000, respectively), in addition to the deep plexus of the superior and temporal sectors (*p* = 0.028 and *p* = 0.001, respectively). The results of the diameter of NVC capillaries and pachyvessels and the CCT and FAZ in CSC groups and HCs are presented in Figure 5.

**Figure 6:**
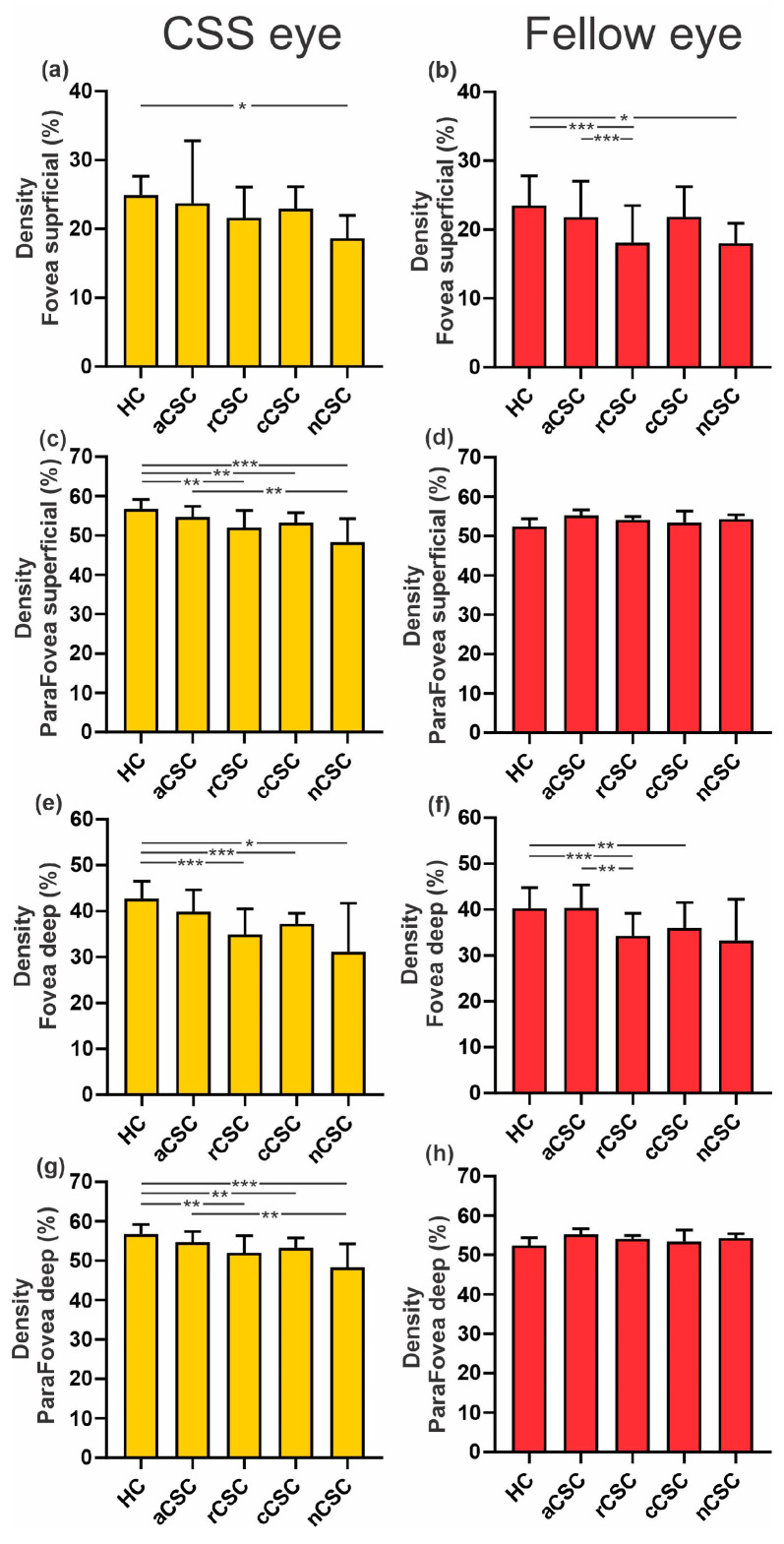
Comparison of vessel density of superficial and deep plexus between CSC eye and fellow eye among patients with CSC and healthy controls (HCs): * *p* < 0.05, ** *p* < 0.01, *** *p* < 0.001. (**a**,**b**) vessel density of the superficial plexus of the fovea of the affected eye and fellow eye, (**c**,**d**) vessel density of the superficial plexus of the parafovea of the affected eye and fellow eye, (**e**,**f**) vessel density of the deep plexus of the fovea of the affected eye and fellow eye, (**g**,**h**) vessel density of the deep plexus of the parafovea of the affected eye and fellow eye.

A comparison of the retinal VD of the superficial and deep plexus of the affected and fellow eye among patients with CSC and control groups is presented in Figure 6. 

#### 3.2.2. Correlations between NVC and Functional Results, OCT Features and General Risk Factors

The diameter of nailfold capillaries correlated significantly with SRF height, EZ disturbances and CRT (*p* = 0.003, *p* = 0.007 and *p* = 0.018, respectively). The NVC pattern was significantly correlated with gender, BCDVA and BCNVA, SRF height, CRT and HF (*p* = 0.000 for all variables). Ramified capillaries and neoangiogenesis were significantly correlated with BCDVA, SRF height, HF and CRT (*p* = 0.000, *p* = 0.000, *p* = 0.000 and *p* = 0.031). Tortuous capillaries were significantly correlated with BCDVA, RPE alteration, SRF height, EZ disturbances, HF, CRT and smoking (*p* = 0.002, *p* = 0.000, *p* = 0.000, *p* = 0.001, *p* = 0.000, *p* = 0.001 and *p* = 0.040, respectively). Glomerular capillaries were significantly correlated with BCDVA, RPE alteration, HF, EZ disturbances, gender and smoking (*p* = 0.002, *p* = 0.003, *p* = 0.003, *p* = 0.001, *p* = 0.003 and *p* = 0.022, respectively). The dilated apical part of capillaries was significantly correlated with gender, BCDVA, SRF height and CRT (*p* = 0.30, *p* = 0.013, *p* = 0.000 and *p* = 0.000, respectively). Meandering capillaries was significantly correlated with SRF height, HF, CRT and smoking (*p* = 0.000, *p* = 0.000, *p* = 0.027 and *p* = 0.038). Freezing hands reported by patients was significantly correlated with HF and EZ disturbances (*p* = 0.043 and *p* = 0.012).

#### 3.2.3. Correlation between Retinal Vessel Density (VD) of Superficial and Deep Plexus, FAZ, Central Choroid Thickness and Diameter of Choroid Pachyvessel Lumen in CSC Patients and Functional Results, OCT Features and General Risk Factors

The diameter of pachyvessel lumen was significantly correlated with BCDVA, RPE alteration, PED, SRF height, HF, EZ disturbances, stress reported by patients and smoking (*p* = 0.000, *p* = 0.000, *p* = 0.022, *p* = 0.000, *p* = 0.000, *p* = 0.05, *p* = 0.026 and *p* = 0.013, respectively). The FAZ was significantly correlated with BCDVA, RPE alteration, CRT and smoking (*p* = 0.001, *p* = 0.048, *p* = 0.034 and *p* = 0.017, respectively). The CCTs of the affected and fellow eye were significantly correlated with BCDVA, HF, CRT, gender and smoking (CSC eye vs. fellow eye *p* = 0.028 vs. *p* = 0.025, *p* = 0.000 vs. *p* = 0.000, *p* = 0.001 vs. *p* = 0.024, *p* = 0.005 vs. *p* = 0.001, and *p* = 0.033 vs. *p* = 0.003, respectively). The relationships of the VD of the superficial and deep plexus of all sectors of affected and fellow eyes are presented in Table 2.

#### 3.2.4. Comparison of the Diameter of Pachyvessel Lumen between Healthy Controls and CSC Groups

The mean diameter of the pachyvessel lumen was significantly higher in all CSC patients compared to controls (*p* = 0.000). The detailed results of that parameter were compared among CSC groups and there were no significant differences. However, the highest results were obtained in rCSC and cCSC, while smaller diameters were found in aCSC and nCSC (240.34 ± 60.61 µm and 239.37 ± 41.50 µm versus 225.60 ± 47.26 µm and 232.27 ± 30.75 µm, respectively). 

#### 3.2.5. Analysis of Risk Factors

In the logistic regression analysis, only tortuous capillaries were an independent risk factor for aCSC, rCSC and cCSC. Additionally, glomerular capillaries were an independent risk factor for cCSC. All revealed risk factors are presented in Table 3.

## 4. Discussion

An abnormal NVC pattern was significantly more common in all CSC groups and among them most common in acute CSC. Additionally, there were strong correlations between some parameters of the NVC examination and the thickness of the central choroid. Moreover, the relationships between NVC results and the FAZ, the lumen diameter of the pachyvessel, and the retinal VD of the superficial and deep plexus of the affected and fellow eye may support the hypothesis of general microvascular abnormalities in CSC patients. Recently, this hypothesis has been widely presented by Spaide et al. [3]. They suggested that some of the pathophysiological processes described in CSC and other pachychoroid spectrum diseases seem to mirror those of chronic venous insufficiency common in other organs [3]. The symptoms of chronic venous insufficiency include spider veins and telangiectasias and dilated blood vessels visible through the skin, especially on the back of the leg [3]. The NVC examination allows the observation and evaluation of small vessels in the skin of the distal parts of the body that are known to instantly respond to changes in microcirculation. This is possible because the capillaries of the finger nailfold run parallel not perpendicular to the skin’s surface, so their structure can be seen. Moreover, structural microcirculation disorders found in the NVC examination are one of the critical features of dermatological and rheumatological diseases, as well as diabetes and hypertension [16]. Both hypertension and diabetes are known risk factors for CSC. For this reason, we excluded patients with diabetes and moderate to severe hypertension from the evaluation of the NVC study to reduce the possible impact of these conditions on our results. In the study group, mild hypertension was reported by two patients with aCSC, four with cCSC, four with rCSC and none with nCSC. 

Disorders of the autoregulation of blood flow through the choroidal vessels are thought to play an essential role in the pathogenesis of CSC. Piccolino et al. suggested that the autoregulation of choroidal vessels in response to systemic hemodynamic fluctuations could be dysregulated in CSC patients [17]. In the states of increased perfusion, they have an increase in blood flow in the choriocapillaries compared to the control group, which suggests a disorder of regulatory mechanisms [17]. Increased venous pressure caused by increased flow or outflow obstruction can lead to venous dilation and fluid overload in the choroid. This, in turn, can cause several effects, including the expression of integrins, MMPs, and cytokines, resulting in dilation of the venous wall, both by excessive fluid loading and remodeling [3]. Pathological changes in the affected tissue include a reduction in the number of capillaries and an increase in their length and diameter [3]. Nicolo et al. showed a reduced vascular flow area in the choriocapillaris but increased blood flow in Sattler’s and Haller’s layers in CSC using swept-source OCT-angiography [9]. Spaide et al. found intervortex venous anastomoses in all pachychoroid patients, which in CSC were mainly located in the central macula [3]. Therefore, we detected and measured the diameter of the pachyvessel, which is suggested to be an anastomotic venous vessel. The results were compared with the lumen diameters of the largest visible choroidal vessel in the control group. The mean diameters of these vessels were significantly higher in the CSC group in comparison to the healthy controls and were significantly correlated with an abnormal NVC pattern, the diameter of nailfold capillaries, ramified capillaries and neoangiogenesis, and tortuous, glomerular and meandering capillaries of the NVC examination. There was no significant difference in the mean diameter of the pachyvessel lumen among CSC groups in contrast to the diameter of nailfold capillaries in the NVC examination. There was a significant difference between healthy controls and all CSC patients, and among CSC groups between chronic and recurrent CSC compared to acute CSC. Interestingly, there was a significant correlation between the diameter of nailfold capillaries and the diameter of the pachyvessel lumen. 

Perhaps the revealed relationships between the parameters of the NVC examination and choroid in addition to retinal circulation in patients with CSC may allow a look at the pathogenesis of CSC from a broader perspective. In addition, the findings may support a hypothesis of the systemic nature of microcirculation disorders in the nailfold and choroid in CSC patients, which requires additional research. 

It is known that the capillaroscopic pattern in healthy individuals may have large variability in results, which often leads to confusion about the overall evaluation between normal and abnormal patterns. Although significant variability can be observed in healthy populations, neoangiogenesis, glomerular and meandering capillaries and dilated apical parts of capillaries are not commonly observed in healthy individuals. The NVC parameters represent characteristic microcirculatory abnormalities and vascular dysregulation due to impaired blood flow autoregulation. Thus, it seems possible that abnormalities in the digital capillaries of the finger nailfold in CSC patients result from vasoconstriction in the microcirculation and suggest a tendency toward vasoconstriction in this disease. That may be supported by the fact that hand freezing, considered one of the symptoms of vascular disorders, was reported only by CSC patients. 

Spaide et al. recently suggested that CSC might be associated with choriocapillaris abnormalities independently of choroidal thickness but relative to pathophysiologic similarities with chronic venous insufficiency [3,18]. Even though this disease is a widespread illness in humans, no patient reported circulatory disorders in our study. However, one of the known risk factors for circulatory disorders, cigarette smoking, was significantly associated with rCSC, cCSC and nCSC. Moreover, significant correlations between smoking and CCT of both eyes of CSC patients, the VD of the superficial and deep plexus of the fovea area of both eyes of CSC patients, and the VD of the superficial plexus of all tested areas in the unaffected eye of CSC patients were found. A significant relationship was also found with the diameter of the pachyvessel lumen, contrary to the diameter of the nailfold capillaries. Smoking was also significantly correlated with BCDVA, the FAZ and meandering, tortuous and glomerular capillaries in the NVC examination. Previously, we also found a significant positive correlation between smoking cigarettes, tortuous capillaries and megacapillaries [13]. 

Moreover, stress, considered one of the significant risk factors for CSC evolution, differed significantly between the control and aCSC and cCSC groups, despite that the control group was gathered during the COVID-19 pandemic, which could have affected the obtained result. Other known risk factors such as H. pylori infection, medication use (including steroids, xylometazoline and phosphodiesterase inhibitors), shift work and sleep disorders were reported by a minority of the patients and had no statistical value.

All NVC results significantly associated with all examined CSC groups were correlated strongly with BCDVA. Additionally, NVC pattern, ramified capillaries and neoangiogenesis, and tortuous capillaries were strongly correlated with SRF height, HF and CRT. Glomerular capillaries and dilated apical parts of capillaries were significantly associated with EZ disturbances and RPE alteration. The first was significantly correlated with HF, and the second with SRF height and CRT. The correlation with HF seems to be interesting. HF may be due to the accumulation of photoreceptors and RPE fluorophores within macrophages during the active stage of CSC [19]. They are considered to be associated with longer persistence of the subretinal fluid, poorer best-corrected visual acuity and the need for treatment in CSC [19]. In our study, their incidence did not differ among CSC patients. However, Lee et al. observed an interesting relationship between the baseline number of HF at the leakage site and the recurrence of CSC [20]. 

It also seems essential that glomerular capillaries are more frequent in cCSC cases compared to the acute and recurrent forms. Moreover, the higher incidence of aneurysmal dilations in nCSC patients compared to those with acute and chronic conditions may indicate their diagnostic value in differentiating subjects predisposed to choroidal neovascularization. However, to our knowledge, no studies compared NVC results with choroidal parameters available on OCT and OCT-A in patients with different types of CSC. 

In 2016, Erol et al. presented the results of NVCs in patients with CSC [12]. They revealed a common incidence of capillary ectasia, aneurysm, microhemorrhage, avascular area, tortuosity, neoformation, bizarre capillaries, bushy capillaries, meandering capillaries and extravasation. Despite this, they found no significant association between NVC results, choroidal thickness and the type of CSC (acute or chronic) [12]. In our previous study, we also found a significantly more common occurrence of abnormal NVC patterns, dilation of capillaries, disorganization of the capillary architecture, ramified capillaries and neoangiogenesis, glomerular capillaries and tortuous capillaries in CSC patients. However, we did not correlate the NVC results with CCT or the lumen of the pachyvessel [13].

It seems that the NVC examination may provide additional information supplementing the assessment of the general condition of a patient with CSC. We believe that the value of the NVC abnormalities can be assessed as another prognostic indicator of CSC progression. With better progress in understanding the pathomechanism of CSC, we may be better able to recognize the signs of higher risk and assess the patient’s condition appropriately. Therefore, it cannot be ruled out that the demonstration of specific changes in the NVC examination may be additional information to optimize treatment plans for patients with CSC. 

There are some limitations of the study. The CSC patients were compared to a healthy control group without any general disorders, including general hypertension and diabetes mellitus, which could influence the NVC examination results. Additionally, the information on the general condition of patients was obtained only from medical history, which may make them underscored.

In this study, abnormalities in nailfold capillaroscopy were more common in CSC patients and differed according to the ocular and general factor profile. Moreover, some appear to be significantly more frequent in certain types of CSC. For example, meandering capillaries were more common in acute and recurrent CSC, glomerular capillaries in chronic CSC and aneurysmal dilations of capillaries in neovascular CSC. In addition, the dilated apical parts of capillaries, abnormal NVC patterns, ramified capillaries and neoangiogenesis were significantly more common in acute CSC.

## 5. Conclusions

The observed digital microcirculation abnormalities in the NVC examination in patients with CSC, such as dilation, meandering, tortuosity and glomerular, may suggest that CSC is a part of systemic micro-vasculopathy. The potential role of the NVC examination in assessing the prognosis of CSC requires further evaluation.

## Figures and Tables

**Table 1:** Demographics and clinical data of central serous chorioretinopathy (CSC) patients and healthy controls (HCs).

	Total	Missing	Control (a)	Acute aCSC (b)	Recurrent rCSC (c)	Chronic cCSC (d)	MNV nCSC (e)	*p* < 0.05
Number of patients (n, %)	193 (100.00)	0	41 (21.24)	43 (22.28)	54 (27.98)	44 (22.80)	11 (5.70)	0.988
Age	193 (100.00)	0	47.12 ± 0.76	46.30 ± 0.90	46.61 ± 0.94	47.50 ± 1.01	47.45 ± 2.08	0.901
Sex, male	147 (76.17)	0	29 (70.73)	35 (81.4)	43 (79.63)	31 (70.45)	9 (81.82)	0.988
Residence area, urban	118 (61.14)	0	27 (65.85)	20 (46.51)	31 (57.41)	31 (70.45)	9 (81.82)	0.613
Stress, y	50 (25.91)	0	18 (43.9)	8 (18.6)	15 (27.78)	6 (13.64)	3 (27.27)	0.020
	*p*_(a-b)_ *; *p*_(a-c)_ ns; *p*_(a-d)_ **; *p*_(a-e)_ ns; *p*_(b-c)_ ns; *p*_(b-d)_ ns; *p*_(b-e)_ ns; *p*_(c-d)_ ns; *p*_(c-e)_ ns; *p*_(d-e)_ ns							
Smoking history, y	40 (20.73)	1 (0.52)	0	7 (16.28)	15 (27.78)	15 (34.09)	3 (27.27)	0.018
	*p*_(a-b)_ **; *p*_(a-c)_ ***; *p*_(a-d)_ ***; *p*_(a-e)_ ***; *p*_(b-c)_ ns; *p*_(b-d)_ ns; *p*_(b-e)_ ns; *p*_(c-d)_ ns; *p*_(c-e)_ ns; *p*_(d-e)_ ns							
*Optical coherence tomography (OCT) variables (n, %)*								
RPE alteration, y	112 (58.03)	1 (0.52)	0 (0.00)	14 (32.56)	52 (98.11)	37 (84.09)	9 (81.82)	0.000
	*p*_(a-b)_ ***; *p*_(a-c)_ ***; *p*_(a-d)_ ***; *p*_(a-e)_ ***; *p*_(b-c)_ ***; *p*_(b-d)_ ***; *p*_(b-e)_ **; *p*_(c-d)_ *; *p*_(c-e)_ *; *p*_(d-e)_ ns							
PED, y	26 (13.47)	1 (0.52)	0	3 (6.98)	12 (22.64)	10 (22.73)	1 (9.09)	0.004
	*p*_(a-b)_ ns; *p*_(a-c)_ **; *p*_(a-d)_ **; *p*_(a-e)_ ns; *p*_(b-c)_ *; *p*_(b-d)_ *; *p*_(b-e)_ ns; *p*_(c-d)_ ns; *p*_(c-e)_ ns; *p*_(d-e)_ ns							
HF, y	108 (55.96)	10 (5.18)	0	27 (65.85)	37 (80.43)	35 (79.55)	9 (81.82)	0.000
	*p*_(a-b)_ ***; *p*_(a-c)_ ***; *p*_(a-d)_ ***; *p*_(a-e)_ ***; *p*_(b-c)_ ns; *p*_(b-d)_ ns; *p*_(b-e)_ ns; *p*_(c-d)_ ns; *p*_(c-e)_ ns; *p*_(d-e)_ ns							
EZ disturbances, y	48 (24.87)	0	1 (2.44)	6 (13.95)	18 (33.33)	17 (38.64)	6 (54.55)	0.004
	*p*_(a-b)_ *; *p*_(a-c)_ **; *p*_(a-d)_ ***; *p*_(a-e)_ ***; *p*_(b-c)_ *; *p*_(b-d)_ **; *p*_(b-e)_ **; *p*_(c-d)_ ns; *p*_(c-e)_ ns; *p*_(d-e)_ ns							
*Nailfold videocapillaroscopy (NVC*) *parameters (n, %)*								
NVC pattern, abnormal	68 (35.23)	0	0	24 (55.81)	22 (40.74)	15 (34.09)	7 (63.64)	0.000
	*p*_(a-b)_ ***; *p*_(a-c)_ ***; *p*_(a-d)_ ***; *p*_(a-e)_ ***; *p*_(b-c)_ ns; *p*_(b-d)_ *; *p*_(b-e)_ ns; *p*_(c-d)_ ns; *p*_(c-e)_ ns; *p*_(d-e)_ ns							
Disorganization of the capillary architecture, y	54 (27.9)	0	5 (12.20)	19 (44.19)	18 (33.33)	8 (18.18)	4 (36.36)	0.176
Hands freezing, y	27 (13.99)	2 (1.04)	0	15 (34.88)	8 (14.81)	0	4 (36.36)	0.000
	*p*_(a-b)_ ***; *p*_(a-c)_ **; *p*_(a-d)_ ns; *p*_(a-e)_ ***; *p*_(b-c)_ *; *p*_(b-d)_ ***; *p*_(b-e)_ ns; *p*_(c-d)_ **; *p*_(c-e)_ ns; *p*_(d-e)_ ***							
Dilated apical part of capillaries, y	48 (24.87)	0	0	18 (41.86)	17 (31.48)	12 (27.27)	1 (9.09)	0.010
	*p*_(a-b)_ ***; *p*_(a-c)_ ***; *p*_(a-d)_ ***; *p*_(a-e)_ ns; *p*_(b-c)_ ns; *p*_(b-d)_ ns; *p*_(b-e)_ *; *p*_(c-d)_ ns; *p*_(c-e)_ ns; *p*_(d-e)_ ns							
Dilated capillaries (diameter > 14 um), y	81 (41.7)	1 (0.52)	9 (21.95)	23 (53.49)	28 (51.85)	17 (38.64)	4 (36.36)	0.143
Ramified capillaries and neoangiogenesis, y	70 (36.27)	2 (1.04)	2 (4.88)	25 (58.14)	22 (42.31%)	16 (36.36)	5 (45.45)	0.000
	*p*_(a-b)_ ***; *p*_(a-c)_ ***; *p*_(a-d)_ ***; *p*_(a-e)_ ***; *p*_(b-c)_ ns; *p*_(b-d)_ *; *p*_(b-e)_ ns; *p*_(c-d)_ ns; *p*_(c-e)_ ns; *p*_(d-e)_ ns							
Tortuous capillaries, y	98 (50.78)	0	3 (7.32)	25 (58.14)	37 (68.52)	25 (56.82)	8 (72.73)	0.000
	*p*_(a-b)_ ***; *p*_(a-c)_ ***; *p*_(a-d)_ ***; *p*_(a-e)_ ***; *p*_(b-c)_ ns; *p*_(b-d)_ ns; *p*_(b-e)_ ns; *p*_(c-d)_ ns; *p*_(c-e)_ ns; *p*_(d-e)_ ns							
Glomerular capillaries, y	59 (30.57)	0	1 (2.44)	8 (18.60)	17 (31.48)	25 (56.82)	8 (72.73)	0.000
	*p*_(a-b)_ *; *p*_(a-c)_ ***; *p*_(a-d)_ ***; *p*_(a-e)_ ***; *p*_(b-c)_ ns; *p*_(b-d)_ ***; *p*_(b-e)_ ***; *p*_(c-d)_ *; *p*_(c-e)_ *; *p*_(d-e)_ ns							
Aneurysmal dilatations, y	18 (9.33)	0	0	3 (6.98)	8 (14.81)	3 (6.82)	4 (36.36)	0.002
	*p*_(a-b)_ ns; *p*_(a-c)_ *; *p*_(a-d)_ ns; *p*_(a-e)_ ***; *p*_(b-c)_ ns; *p*_(b-d)_ ns; *p*_(b-e)_ **; *p*_(c-d)_ ns; *p*_(c-e)_ ns; *p*_(d-e)_ **							
Broken capillaries, y	22 (11.40)	0	15 (36.59)	0	3 (6.82)	2 (3.70)	3 (27.27)	0.000
	*p*_(a-b)_ ***; *p*_(a-c)_ ***; *p*_(a-d)_ ***; *p*_(a-e)_ ns; *p*_(b-c)_ ns; *p*_(b-d)_ ns; *p*_(b-e)_ ***; *p*_(c-d)_ ns; *p*_(c-e)_ *; *p*_(d-e)_ **							
Microhemorrhages, y	42 (21.76)	0	9 (21.95)	10 (23.26)	15 (27.78)	6 (13.64)	2 (18.18)	0.981
Megacapillaries, y	6 (3.11)	0	0	2 (4.65)	0	2 (4.55)	2 (18.18)	0.285
Meandering capillaries, y	55 (28.50)	0	3 (7.32)	18 (41.86)	22 (40.74)	8 (18.18)	4 (36.36)	0.035
	*p*_(a-b)_ ***; *p*_(a-c)_ ***; *p*_(a-d)_ ns; *p*_(a-e)_ *; *p*_(b-c)_ ns; *p*_(b-d)_ *; *p*_(b-e)_ ns; *p*_(c-d)_ *; *p*_(c-e)_ ns; *p*_(d-e)_ ns							
Bizarre capillaries, y	14 (7.25)	0	1 (2.44)	2 (4.65)	9 (16.67)	2 (4.55)	0	0.414

Abbreviation: RPE—retinal pigment epithelium, PED—pigment epithelium detachment, HF—hyper-reflective foci, EZ—ellipsoid zone, * *p* < 0.05, ** *p* < 0.01, *** *p* < 0.001, ns—not significant.

**Table 2:** Correlation between retinal vessel density (VD) of superficial and deep plexus of examined sectors in affected and fellow eye of CSC patients and functional results, OCT features and general risk factors. Spearman’s coefficients (r), the value of *p* is presented in parentheses. The significant coefficients are marked with an asterisk (*p* < 0.05).

Variables	Sectors of VD							
	CSC Eye				Fellow Eye			
*Superficial Plexus*								
Factor r (*p*)	Superior	Inferior	Temporal	Nasal	Superior	Inferior	Temporal	Nasal
BCDVA	0.290 * (0.000)	0.172 * (0.017)	0.211 * (0.003)	0.248 * (0.001)	0.205 * (0.004)	0.116 (0.111)	0.164 * (0.024)	0.188 * (0.010)
RPE alteration	−0.243 * (0.010)	−0.183 * (0.011)	−0.217 * (0.002)	−0.227 * (0.001)	−0.177 * (0.015)	−0.103 (0.157)	−0.113 (0.121)	−0.151 * (0.039)
HF	−0.133 (0.073)	−0.123 (0.097)	−0.097 (0.191)	−0.135 (0.067)	−0.122 (0.101)	−0.073 (0.328)	−0.073 (0.328)	−0.097 (0.197)
EZ disturbances	0.168 * (0.019)	0.081 (0.258)	0.154 * (0.032)	0.185 * (0.010)	0.189 * (0.009)	0.111 (0.128)	0.090 (0.217)	0.166 * (0.023)
CRT	0.142 * (0.050)	0.135 (0.062)	0.157 * (0.030)	0.189 * (0.009)	0.144 * (0.049)	0.127 (0.083)	0.080 (0.277)	0.164 * (0.026)
Gender	−0.124 (0.085)	−0.119 (0.097)	−0.170 * (0.018)	−0.008 (0.907)	−0.098 (0.174)	−0.062 (0.394)	−0.167 * (0.022)	−0.144 * (0.048)
Smoking	−0.134 (0.064)	−0.118 (0.101)	−0.101 (0.162)	−0.191 * (0.008)	−0.249 * (0.001)	−0.201 * (0.006)	−0.246 * (0.001)	−0.250 * (0.001)
*Deep Plexus*								
BCDVA	0.161 * (0.027)	0.295 * (0.000)	0.305 * (0.000)	0.263 * (0.000)	0.019 (0.790)	−0.116 (0.109)	−0.015 (0.829)	−0.042 (0.562)
RPE alteration	−0.106 (0.148	−0.303 * (0.000)	−0.300 * (0.000)	−0.246 * (0.001)	−0.050 (0.485)	0.085 (0.240)	0.067 (0.354)	0.006 (0.932)
HF	−0.075 (0.318)	−0.196 * (0.009)	−0.170 * (0.023)	−0.147 * (0.050)	−0.010 (0.891)	0.113 (0.128)	−0.045 (0.542)	0.041 (0.574)
CRT	0.973 (0.187)	0.060 (0.418)	0.136 (0.064)	0.163 * (0.026)	0.066 (0.361)	0.062 (0.391)	0.081 (0.265)	0.064 (0.376)
EZ disturbances	−0.030 (0.678)	0.150 * (0.040)	0.191 * (0.008)	0.109 (0.136)	0.086 (0.235)	−0.038 (0.602)	0.058 (0.423)	0.036 (0.619)
Gender	−0.102 (0.163)	0.081 (0.266)	0.034 (0.640)	−0.088 (0.231)	−0.082 (0.254)	−0.062 (0.393)	−0.135 (0.060)	−0.181 * (0.012)
Smoking	−0.044 (0.548)	−0.114 (0.118)	−0.075 (0.304)	−0.139 (0.057)	−0.014 (0.847)	0.039 (0.589)	−0.039 (0.592)	0.007 (0.918)

Abbreviation: RPE—retinal pigment epithelium, CRT—central retinal thickness, HF—hyper-reflective foci, EZ—ellipsoid zone.

**Table 3:** Analysis of risk factors for CSC types.

**Analysis of Risk Factors for aCSC**				
Fit statistics				
Hosmer–Lemeshow test (*p* value)		0.409		
R2 Cox–Snell		0.559		
R2 Nagelkerka		0.745		
AIC		57.009		
Risk Factor	β	Wlad χ^2^	*p* values	OR
Intercept	−9.369	1.261	0.2614	
VD parafovea deep fellow eye	0.387	8.575	0.0034	1.472
VD parafovea deep	−0.353	7.384	0.0065	0.702
Diameter pachyvessel	0.037	11.839	0.0006	1.038
Tortuous capillary; yes (1)	4.144	11.742	0.0006	63.092
**Analysis of Risk Factors for rCSC**				
Fit statistics				
Hosmer–Lemeshow test (*p* value)		0.457		
R2 Cox–Snell		0.663		
R2 Nagelkerka		0.888		
AIC		37.189		
Risk Factor	β	Wlad χ^2^	*p* values	OR
Intercept	32.822	3.640	0.0563	
Diameter pachyvessel	0.045	12.906	0.0003	1.046
VD superior superficial fellow eye	−0.629	5.491	0.0191	0.533
VD parafovea deep	−0.582	7.840	0.0051	0.559
VD parafovea deep fellow eye	0.455	5.003	0.0253	1.576
Tortuous capillary; yes (1)	2.135	0.681	0.0017	71.498
**Analysis of Risk Factors for cCSC**				
Fit statistics				
Hosmer–Lemeshow test (*p* value)		0.963		
R2 Cox–Snell		0.668		
R2 Nagelkerka		0.891		
AIC		33.222		
Risk Factor	β	Wlad χ^2^	*p* values	OR
Intercept	27.873	5.481	0.0192	
Diameter pachyvessel	0.049	6.755	0.0093	1.051
VD parafovea deep	−0.551	6.077	0.0137	0.576
VD fovea deep fellow eye	−0.142	3.402	0.0650	0.867
Glomerular capillary; yes (1)	2.064	5.747	0.0165	62.100
Tortuous capillary; yes (1)	2.179	5.372	0.0204	78.137
**Analysis of Risk Factors for nCSC**				
Fit statistics				
Hosmer–Lemeshow test (*p* value)		0.993		
R2 Cox–Snell		0.592		
R2 Nagelkerka		0.919		
AIC		13.056		
Risk Factor	β	Wlad χ^2^	*p* values	OR
Intercept	47.903	4.612	0.0317	
VD nasal deep	−1.239	6.015	0.0142	0.2895
CCT	0.048	3.863	0.0493	1.0497

## Data Availability

The data presented in this study are available on request from the corresponding author. The data are not publicly available due to the privacy restriction.
